# TTBK1 and CK1 inhibitors restore TDP-43 pathology and avoid disease propagation in lymphoblast from Alzheimer’s disease patients

**DOI:** 10.3389/fnmol.2023.1243277

**Published:** 2023-08-09

**Authors:** Loreto Martinez-Gonzalez, Eva P. Cuevas, Carlota Tosat-Bitrián, Vanesa Nozal, Carmen Gil, Valle Palomo, Ángeles Martín-Requero, Ana Martinez

**Affiliations:** ^1^Centro de Investigaciones Biológicas “Margarita Salas”-CSIC, Madrid, Spain; ^2^Centro de Investigación Biomédica en Red en Enfermedades Neurodegenerativas (CIBERNED), Instituto de Salud Carlos III, Madrid, Spain; ^3^Instituto Madrileño de Estudios Avanzados en Nanociencia (IMDEA-Nanociencia), Madrid, Spain

**Keywords:** TDP-43 pathology, Alzheimer’s disease, CK1 inhibitor, TTBK1 inhibitor, drug discovery

## Abstract

**Introduction:**

TDP-43 proteinopathy in Alzheimer’s disease (AD) patients is recently emerging as a relevant pathomolecular event that may have been overlooked. Recent results in immortalized lymphocytes from AD patients have shown not only an increase of post-translational modifications in TDP-43, such as hyperphosphorylation and fragmentation, but also its prionic behaviour and cell-to-cell disease transmission. With the main goal to advance therapeutic interventions, we present in this work different kinase inhibitors with potential to restore this pathological mechanism.

**Methodology:**

We have used immortalized lymphocytes from healthy controls and AD severe patients to evaluate the correction of TDP-43 pathology after the treatment with previously synthetized TTBK1 and CK1 inhibitors. Moreover we used the conditioned mediums of these cells to perform different disease propagation experiments.

**Results:**

TDP-43 pathology observed in lymphoblasts from severe AD patients is reduced after the treatment with TTBK1 and CK1 inhibitors (decreasing phosphorylation and increasing nuclear localisation), Furthermore, the significant increase in TDP-43 phosphorylation, cytoplasmic accumulation and aberrant F-actin protrusions (TNT-like structures) observed in control cells growing in CM from AD lymphoblasts were abolished when the CM from AD lymphoblasts treated with previously reported TTBK1 and CK1 inhibitors were used. In addition, the cytosolic transport mediated by molecular motors of the receptor cells was altered with the induced TDP-43 pathology, but it was not produced with the abovementioned pretreated CMs.

**Conclusion:**

TTBK1 and CK1 inhibitors, specially VNG1.47 and IGS2.7 compounds, restore TDP-43 pathology and avoid cell-to-cell propagation in immortalized lymphocytes from AD patients, being excellent candidates for the future therapy of this prevalent and devastating disease.

## 1. Introduction

Alzheimer’s disease (AD) is the most common form of dementia accounting for more than 35 million people affected worldwide. It is considered a chronic illness and also a highly concerning world health problem. As the main risk factor is age and different studies predict an increase of the life expectancy of the population, an important escalation of the number of AD patients is expected in the following years (Alzheimer’s Association Report, 2022). In this dementia, the cholinergic neurons in the hippocampus die progressively causing different symptoms such as: cognitive impairment, learning difficulties and dependence (Lane et al., 2018). Diverse pathological events at the molecular level have been described, being the most prevalent hypothesis for the degradation of these neurons the accumulation of intracellular neurofibrillary tangles formed by hyperphosphorylated protein tau and extracellular senile plaques composed of β-amyloid fragments (Ashford, 2019). Although these pathological events have been known for decades, their direct modulation using different approaches has not yet provided an effective treatment able to stop the progression of the disease. Within the available drugs to alleviate the symptoms of AD we can differentiate the inhibitors of acetylcholinesterase (tacrine, donepezil, galantamine and rivastigmine) and the receptor antagonist of *N*-methyl-D-aspartate (memantine) (Yiannopoulou and Papageorgiou, 2020).

In the last years, a new protein has attracted the focus in the field of neurodegenerative diseases: transactive response DNA-binding protein of 43 kDa (TDP-43). Hyperphosphorylated TDP-43 was identified as the main component of ubiquitinated protein aggregates found in amyotrophic lateral sclerosis (ALS) and frontotemporal dementia (FTLD) patients (Neumann et al., 2006). Since then, different studies have assessed the pathological role of this key cellular regulator in other diseases such as: Hungtington’s disease, Alexander’s disease, and progressive supranuclear palsy among others (Schwab et al., 2008; Palomo et al., 2019).

Particularly in AD, several studies have determined the impact of the deposition of TDP-43 in patients’ brain, stablishing that it aggravates the memory loss and the hippocampal atrophy. Furthermore, TDP-43 pathology has been found in half of the patients that were considered to havepure Alzheimer’s disease (James et al., 2016; Meneses et al., 2021). Meanwhile in the last years a new brain disorder, called limbic-predominant age-related TDP-43 encephalopathy (LATE), that often presents similar symptoms than AD, has been described (Nelson et al., 2019). It is estimated that LATE is responsible for the 15–20% of dementias while around 40% of people with dementia have some TDP-43 encephalopathy in their brains (Besser et al., 2020).

TDP-43 is an important protein involved in the transcription, splicing and transport of numerous RNAs and it is found mainly in the nucleus where it exerts its main functions. However, at physiological conditions, it presents a localisation equilibrium with the cytoplasm, where it can be found in a 5–20% of the total amount of the protein (Woo et al., 2017). In pathological conditions different post-translational modifications alter its dynamic control and solubility. Among these alterations, fragments of 25 and 35 kDa and hyperphosphorylated forms are the most common features of the cytoplasmic aggregates (Neumann et al., 2009; Medina et al., 2014).

Few protein kinases have been described to exert this TDP-43 pathological hyperphosphorylation being casein kinase 1 (CK1), cell division cycle kinase 7 (CDC7), glycogen synthase kinase 3 (GSK3) and tau tubulin kinase 1 (TTBK1) the better characterized (Kametani et al., 2009; Liachko et al., 2013; Moujalled et al., 2013; Tian et al., 2021). Brain permeable selective inhibitors of these kinases emerged as promising therapeutic candidates for TDP-43-pathies (Martinez-Gonzalez et al., 2020, 2021; Rojas-Prats et al., 2021; Nozal et al., 2022). Among thus, inhibitors of TTBK1, a kinase expressed preferentially in central nervous system, and CK1 inhibitors, may offer a great potential for AD treatment. In particular AD with comorbid TDP-43 pathology (Latimer and Liachko, 2021), because of their ability to reduce both tau and TDP-43 phosphorylation *in cellulo* and *in vivo* models (Sundaram et al., 2019; Martinez-Gonzalez et al., 2020; Halkina et al., 2021; Nozal et al., 2022).

Thus, TTBK1 and CK1 are gaining stage as therapeutic targets for neurodegenerative diseases. Some inhibitors have been described and their efficacy in tau and TDP-43 modulation have been reported (Halkina et al., 2021; Nozal et al., 2022; Roth et al., 2022). Among them, the small heterocyclic compounds named IGS2.7 and VNG1.47 have shown to reduce tau and TDP-43 phosphorylation in neuroblastoma cell cultures and to promote the recovery of TDP-43 homeostasis (decrease of hyperphosphorylation and increase in nuclear localisation) in a human cell based model of ALS (Salado et al., 2014; Martinez-Gonzalez et al., 2020; Nozal et al., 2022). They have excellent *in vivo* pharmacokinetic profiles with a 2 to 3:1 ratio regarding brain to plasma penetration and show motor neuron protection and decrease of TDP-43 phosphorylation in the spinal cord of TDP-43-transgenic mice after chronic treatment (Martinez-Gonzalez et al., 2020; Nozal et al., 2022).

In this work, we report how the CK1 inhibitor, named IGS2.7 (Salado et al., 2014), and the TTBK1 inhibitor, called VNG1.47 (Nozal et al., 2022), are able not only to recover the TDP-43 homeostasis in lymphoblastoid cell lines of severe AD patients, but also to stop the cell-to-cell transmission of the TDP-43 pathology in this kind of cells. These results reinforce the potential of both type of protein kinase inhibitors as effective disease modifying agents for the treatment of neurodegenerative diseases, mainly for AD.

## 2. Materials and methods

### 2.1. Materials

RPMI 1640 culture medium (Cat#21875034 Gibco/Thermo Fisher, Waltham, MA, USA), penicillin/streptomycin (Cat#15140-122, Gibco/Thermo Fisher Waltham, MA, USA), Fetal Bovine Serum (FBS) (Cat#: F7524, Merck, Madrid, Spain), polyvinylidene difluoride (PVDF) membranes for Western blots (Bio-Rad, Alcobendas, Madrid, Spain), Chemiluminescence (ECL) system (Amersham, Uppsala, Sweden), Pierce BCA Protein Assay kit (Thermo Fisher, Waltham, MA, USA), protease inhibitor complete mini mixture (Roche, Mannheim, Germany).

Kinase inhibitors IGS2.7 and VNG1.47 were synthetized in our laboratory following previous described procedures (Salado et al., 2014; Nozal et al., 2022). Their chemical structure, IC_50_ values regarding kinase inhibition together with effective permeability values that predict their ability to cross the blood–brain barrier (BBB), are provided in Table 1.

**TABLE 1:** Overview of the compounds used in this study.

Compound	Chemical structure	Target	IC_50_	BBB prediction	*Pe* (10^–6^ cm.S^–1^)
IGS2.7	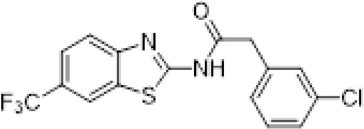	Casein Kinase 1 (CK1)	23 ± 2 nM	CNS +	11.3 ± 2.0
VNG1.47	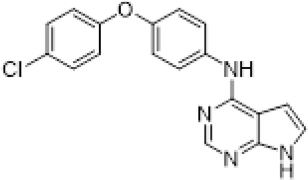	Tau Tubulin Kinase 1 (TTBK1)	0.2 ± 0.1 μM	CNS +	11.5 ± 1

IC_50_: compound concentration able to inhibit the 50% of kinase activity *in vitro. Pe*: apparent Central nervous system permeability determined by parallel artificial membrane permeability assay (PAMPA). Compounds were classified as CNS + (brain penetrant). Values shown are the mean ± SD.

Antibodies used in this study are listed in Table 2. Supplier companies were Santa Cruz Biotechnologies (Santa Cruz, CA, USA), Cell Signal (Danvers, MA, USA), Thermo Fisher (Waltham, MA, USA), Molecular Probes (Thermo Fisher), Bio-Rad (Alcobendas, Madrid, Spain) or Proteintech, (Manchester, UK).

**TABLE 2:** Primary and secondary antibodies used in the analysis.

Primary antibody	Species	Dilution (WB/IF)	Supplier (Catalog#)	RRID
TDP-43	Rabbit	1:1,000/na	Proteintech (10782-2-AP)	AB_615042
TDP-43	Mouse	1:1,000/1:100	Proteintech (67345-1-Ig)	AB_2882603
p(Ser409/410)-TDP-43	Rabbit	1:500/1:1,000	Proteintech (22309-1-AP)	AB_11182943
α-Tubulin	Mouse	1:5,000/na	Santa Cruz (23948)	AB_628410
GAPDH	Rabbit	1:1,000/na	Cell Signal (5174)	AB_10622025
**Secondary antibody**	**Immunological procedure**	**Dilution**	**Supplier (Catalog#)**	
Goat anti-mouseIgG HRP conjugate	WB	1:7,000	Bio-Rad (1706516)	AB_11125547
Goat anti-rabbitIgG HRP conjugate	WB	1:7,000	Bio-Rad (1706516)	AB_567028
anti-mouse Alexa 488	IF	1:1,000	Molecular Probes (A-11001)	SCR_004098

na, not apply; WB, western blot; IF, immunofluorescence.

### 2.2. Subjects and establishment of lymphoblastoid cell lines

Blood samples from two healthy controls and two patients suffering from severe AD were obtained after written informed consent. Patients were diagnosed in the Hospital 12 de Octubre (Madrid, Spain) as severe Alzheimer’s disease patients based on the criteria of the National Institute of Neurological and Communicative Disorders and Stroke and the Alzheimer’s Disease and Related Disorders Association (NINCDS-ADRDA) (McKhann et al., 1984). Classification of severe degree of AD was performed using DSM-III-R criteria. Mental State Examination was used to assess cognitive function (Folstein et al., 1975) Severe AD cases have MMSE score less than 10. Control individuals were patients’ family members who showed no symptoms of neurological disease or cognitive deterioration. All study protocols were approved by the Ethic Committee of Clinical Investigation of the Hospital 12 de Octubre (CEIC02506) and by the Spanish National Research Council Institutional Review Board (15 March 2007).

Peripheral blood mononuclear cells (PBMCs) were isolated on Lymphoprep™ density-gradient centrifugation following the manufacturer’s instructions (Axix-Shield Po CAS, Oslo, Norway). Lymphoblastoid cell lines (LCLs) were established by infecting peripheral blood lymphocytes with the Epstein Barr virus (EBV) as previously described (Hussain and Mulherkar, 2012).

### 2.3. Cell culture

Lymphoblastoid cell lines were grown in suspension in T flasks in an upright position, in RPMI 1640 medium (1 × 10^6^ cells/mL) that contained 1% penicillin/streptomycin and 10% (v/v) fetal bovine serum (FBS). U2OS cells were obtained from the American Type Culture Collection and grown in DMEM media, supplemented with 10% (v/v) fetal bovine serum and 1% penicillin/streptomycin. All cell lines were grown at 37°C in a humidified 5% CO_2_ atmosphere.

### 2.4. Kinase inhibitors treatments

TTBK1 inhibitor VNG1.47 and CK1 inhibitor IGS2.7 were the compounds used in these studies (Table 1). Cells were seeded at initial density of 1 × 10^6^ cells × mL^–1^. 24 h later were exposed to kinase inhibitors at concentrations of 10 μM and 5 μM, respectively, for further 24 h. Then, cells were harvested and processed for Western blotting or immunofluorescence analysis.

### 2.5. Conditioned medium experiments

AD lymphoblastoid cells were treated with or without VNG1.47 (10 μM) or IGS2.7 (5 μM). Conditioned medium was collected after 72–96 h and added to healthy cells in a ratio 3:1 with fresh medium. After 72 h cells were harvested and processed for Western blotting or immunofluorescence analysis.

### 2.6. Cell extracts

Cells were collected by centrifugation, washed with PBS, and then lysed in ice-cold RIPA buffer (50 mM Tris-HCl (pH 7.4), 150 mM NaCl, 5 mM EDTA, 15 mM MgCl_2_, 0.5% (vol/vol) sodium deoxycholate, 0.5% (vol/vol) NP-40 and 0.1% (vol/vol) SDS), containing 1 mM phenylmethylsulfonylfluride (PMSF), 1 mM sodium orthovanadate, 1 mM sodium pyrophosphate and protease inhibitor mixture). Pierce BCA Protein Assay kit was used to determine the protein content of the extracts.

### 2.7. Western blot analysis

Equal amounts of proteins (50 μg) were resolved by SDS–polyacrylamide gel electrophoresis, transferred to polyvinylidene fluoride (PVDF) membrane and blocked with 5% BSA in TTBS 1X. The antibodies used are listed in Table 2. After primary antibodies incubation (4°C, o/n), signals were amplified using species-specific antisera conjugated with horseradish peroxidase. Bands were detected with a chemiluminescent substrate detection system ECL using the Chemidoc Imaging System (Bio-Rad, Alcobendas, Madrid, Spain). Relative band intensities were quantified using Image J software (National Institutes of Health, Bethesda, MD, USA).

### 2.8. Immunofluorescence analysis

Immunofluorescence analysis was performed on cells grown on coverslips. For the attachment of lymphoblastoid cells, the coverslips were previously coated with a solution of 0.025% Gelatin (Sigma, Madrid, Spain) for 30 min at room temperature followed by a solution of 1 mg/mL poly-L-lysine (Sigma, Madrid, Spain) diluted 1:50 in Borax buffer (Na2B4O7 ⋅ 10H2O 15 mM, pH 8.4) overnight at 37°C. Cells were then fixed for 25 min with 4% paraformaldehyde and permeabilized with 0.1% Triton X-100 for 10 min. Then samples were blocked with PBS-1% BSA for 60 min at 37°C.

Cells were incubated with the primary and secondary antibodies described in Table 2 at 37°C in a humidified chamber. Nuclear staining was performed by incubation with DAPI (1:1,000, Sigma) and Alexa Fluor-568 Phalloidin (1:1,000, Molecular Probes, Waltham, MA, USA) was used for F-actin stain. Preparations were mounted with Fluor Save reagent (Calbiochem, Madrid, Spain). High-resolution images were acquired using a confocal microscope Leica TCS SP5 × 100 oil immersion objective. Images were analysed using Leica Application Suite X (version 3.5.7.23225) and Image J software (version 1.53K).

### 2.9. Peptidic probes

Peptidic probes combined a cell penetrating region and a kinesin binding domain and were labelled at the *N*-termini with Cy5 to provide a fluorescent kinesin binding peptide. Their synthesis and labelling has been previously described (Oliva et al., 2022).

### 2.10. Intracellular transport analysis

U2OS cells were seeded on 8-well ibidi plates and treated for 72 h with the conditioned medium from healthy, AD lymphoblasts or with the conditioned medium from lymphoblasts treated with the drug candidates, IGS2.7 (5 μM) and VNG1.47 (10 μM). After the CM treatments, the probe Cy5-KBP was added to the cells at 2.5 μM for 15 min at 37°C. Peptides were washed using DMEM without phenol red and imaged using a confocal laser scanning microscope Leica TCS SP8 with a 63x oil immersion objective that included a humidified incubation chamber, a CO_2_ controller and a heating unit. Cy5 was excited at 646 nm and its fluorescence emission was collected at 660–720 nm. Images were recorded every 1.793 s for 4 min as 2-layer-z-stacks. For each condition, three different fields were imaged in two separate experiments.

Single-particle trajectories were analyzed with the TrackMate plugin from the ImageJ software. Specifically, the LoG detector and the linear motion LAP tracker were used to detect and link particles with an estimated blob diameter of 1 μm and at a maximum distance of 2 μM. More filters were included to discard trajectories with less than 5 spots and with a maximum speed of 1 μm s^–1^. Track mean displacement and track mean velocity were calculated and normalized to control cells in each experiment. Data were analyzed by one-way ANOVA using GraphPad Prism 8 software. For multiple comparisons, Bonferroni’s correction was applied.

### 2.11. Statistical analysis

Statistical analyses were performed using Graph Pad Prism software version 6 (La Jolla, CA, USA). All the statistical data are presented as mean ± standard error of the mean (SEM). Statistical significance was estimated using two-tailed Student’s t-test for statistical comparisons between groups, or one-way ANOVA followed by the Bonferroni test for multiple comparators. A “*p*-value <0.05” was considered statistically significant.

## 3. Results

### 3.1. TTBK1 inhibitor VNG1.47 and CK1 inhibitor IGS2.7 recover TDP-43 homeostasis in lymphoblasts from severe AD patients

**FIGURE 1:**
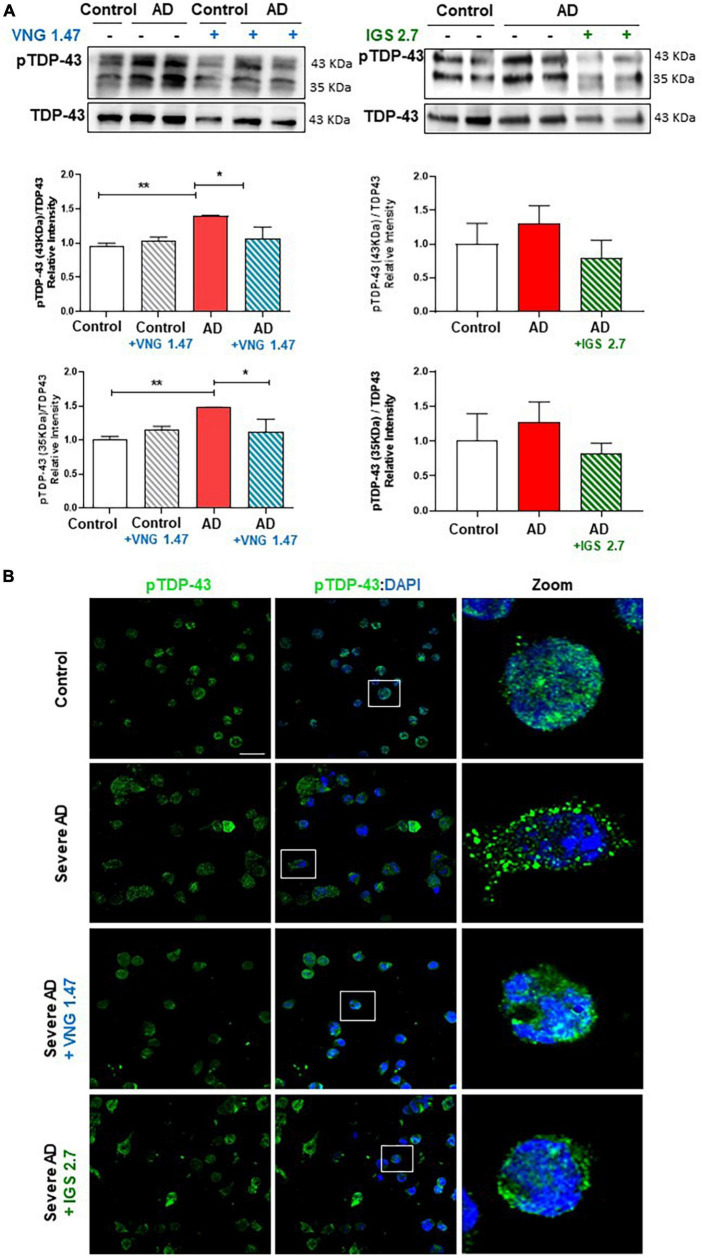
Effect of protein kinase inhibitors in TDP-43 phosphorylation status in severe AD and control lymphoblasts. Cells were incubated in the presence or absence of VNG1.47 (10 μM, TTBK1 inhibitor) or IGS2.7 (5 μM, CK1 inhibitor) for 24 h. **(A)** Representative immunoblots are shown. Densitometric quantification of p-TDP-43 (bands of 35 and 43 kDa) was normalized with total TDP-43 levels. Bars are the mean ± SD for each experimental group of three independent experiments. **(B)** Representative confocal immunofluorescence images of cells stained with anti-TDP-43 antibody (green), F-actin (red) and DAPI (blue) are shown. Scale bar, 20 μm. Magnified cells from images are shown for better visualization. Data were assessed by one-way ANOVA and *post hoc* Bonferroni’s analysis (*p < 0.05, **p < 0.01).

VNG1.47 and IGS2.7 have shown to be effective in the experimental treatment of lymphoblasts from ALS patients which are characterized by presenting TDP-43 hyperphosphorylated cytosolic aggregates, the main pathological hallmark of ALS (Martinez-Gonzalez et al., 2020; Nozal et al., 2022). We have recently reported the presence of TDP-43 pathology in AD immortalized lymphocytes (Cuevas et al., 2022). However, it was not explored whether small molecule protein kinases inhibitors would be able to modulate TDP-43 pathological hallmark in samples from AD patients. Therefore, we assessed the therapeutic effect of two well-characterized protein kinase inhibitors on restoring TDP-43 homeostasis in AD. We treated two healthy controls and two severe AD samples of lymphoblasts with VNG1.47 (10 μM) and IGS2.7 (5 μM) for 24 h. TDP-43 phosphorylation was evaluated by both Western blotting (WB) and immunofluorescence techniques using a phospho-specific (Ser 409/410) anti-TDP-43 antibody (Table 2). As previously described, we found higher levels of TDP-43 hyperphosphorylated in AD lymphoblasts compared to control lymphoblasts from healthy individuals (Figure 1). The increased phosphorylation of TDP-43 both full-length and 35 KDa fragment was significantly reduced when treated with the two compounds (Figure 1A). The effects of VNG1.47 and IGS2.7 were further verified by immunofluorescence analysis (Figure 1B). Overall TDP-43 content in healthy controls and AD patients was unaffected by these treatments.

**FIGURE 2:**
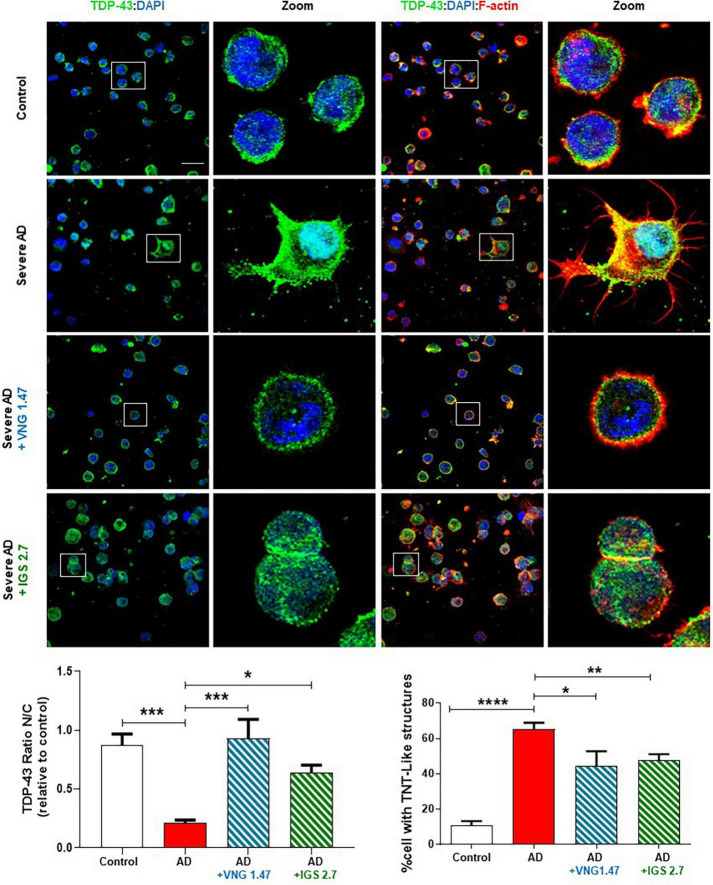
Subcellular localisation of TDP-43 detected by immunofluorescence staining after compounds VNG1.47 (10 μM) and IGS2.7 (5 μM) treatment in severe AD and control lymphoblasts. Representative confocal immunofluorescence images of cells stained with anti-TDP-43 antibody (green), F-actin (red) and DAPI (blue) are shown. Scale bar, 20 μm. Magnified cells from images are shown for better visualization. Fluorescence intensity quantification was performed using Image J software in at least 4 fields of view (A.U, arbitrary units) in 2 severe AD patients and 2 controls. Bars are the mean ± SD of three independent experiments. Graphs represent nucleus-cytoplasm ratio (mean nuclear intensity divided by the mean cytoplasmic intensity) and % of cells with F-actin protrusions (TNT-like structures). Data were assessed by one-way ANOVA and *post hoc* Bonferroni’s analysis (*p < 0.05, **p < 0.01, ***p < 0.001, ****p < 0.0001).

We next investigated whether the reduction of this post-translational modification restores the nucleus-cytoplasmic functional balance of TDP-43 and reduces the aberrant F-actin cytoskeleton protrusions, reminiscent of tunnelling nanotubes or TNT-like structures that characterized these AD lymphoblasts (Cuevas et al., 2022). With this aim, TDP-43 subcellular localisation and cytoskeleton morphology were analysed by immunofluorescence. As expected, lymphoblasts from AD patients had higher cytosolic TDP-43 content and higher amount of TNT-like structures than healthy cells (Figure 2). After 24 h of treatment with VNG1.47 or IGS2.7, TDP-43 recovered subcellular localisation as the nucleus/cytosol ratio was significantly increased in both cases (Figure 2). We also observed a decrease in the number of cells with F-actin protrusions, as treated cells showed less TNT-like structures (Figure 2).

### 3.2. VNG1.47 and IGS2.7 treatment avoids cell-to cell transmission of TDP-43 pathology in lymphoblasts from severe AD patients

The prion-like properties of TDP-43 have been described in several TDP-43-linked diseases. We have recently documented the prionic behaviour of this protein in AD lymphoblasts throughout conditioned medium (CM) experiments which contains a 25 kDa TDP-43’s prionic fragment (Cuevas et al., 2022). To further characterize the effect of these kinase inhibitors in preventing the TDP-43 cell-to-cell transmission, we performed different studies using CM from severe AD cases treated independently with the TTBK1 inhibitor VNG1.47 or CK1 inhibitor IGS2.7.

**FIGURE 3:**
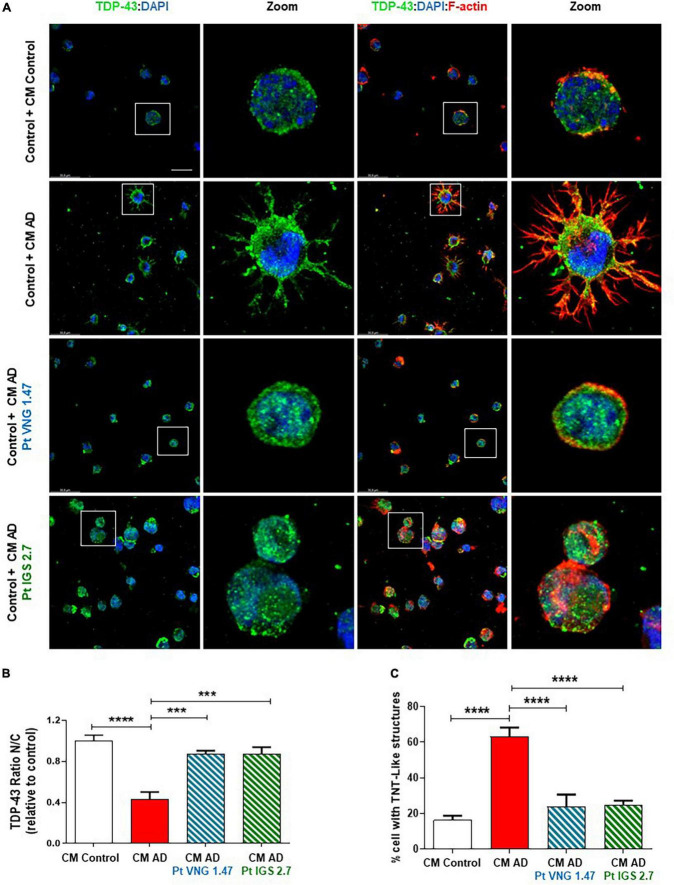
Conditioned medium from AD lymphoblasts pre-treated with kinase inhibitors prevent TDP-43 pathology in healthy cells. Control lymphoblasts were cultured in presence or absence of CM from severe AD cells pre-treated or not with VNG1.47 (10 μM) or IGS2.7 (5 μM) for 72 h. **(A)** Representative confocal immunofluorescence images of cells stained with anti-TDP-43 antibody (green), F-actin (red) and DAPI (blue) are shown. Scale bar, 20 μm. Magnified cells from images are shown for better visualization. Fluorescence intensity quantification was performed using Image J software in at least 4 fields of view (A.U, arbitrary units). Bars are the mean ± SD of three independent experiments. **(B)** Graph represents nucleus-cytoplasm ratio (mean nuclear intensity divided by the mean cytoplasmic intensity). **(C)** Graph represents % of cells with F-actin protrusions (TNT-like structures). Data were assessed by one-way ANOVA and *post hoc* Bonferroni’s analysis (***p < 0.001, ****p < 0.0001).

First, we cultured AD lymphoblasts treated with and without the kinase inhibitors for 72 h. These pre-treated (ptCM) and non-treated (CM) mediums from AD lymphoblasts were collected and added to healthy control lymphoblasts for 72 h. Immunofluorescence staining with an anti-TDP-43, revealed the presence of TDP-43 pathological changes such as cytosolic localisation and generation of TDP-43 aggregates in healthy cells exposed to CM from AD lymphoblasts compared to control cells (healthy cells exposed to CM from healthy lymphoblasts) (Figure 3). However, healthy cells cultured with ptCM from AD patients prevented TDP-43 mislocalization and decreased the protein aggregation (Figure 3). Furthermore, aberrant cytoskeleton F-actin protrusions shown in healthy lymphoblasts exposed to the patient’s medium, were also reduced when cells were cultured with ptCM (Figure 3).

**FIGURE 4:**
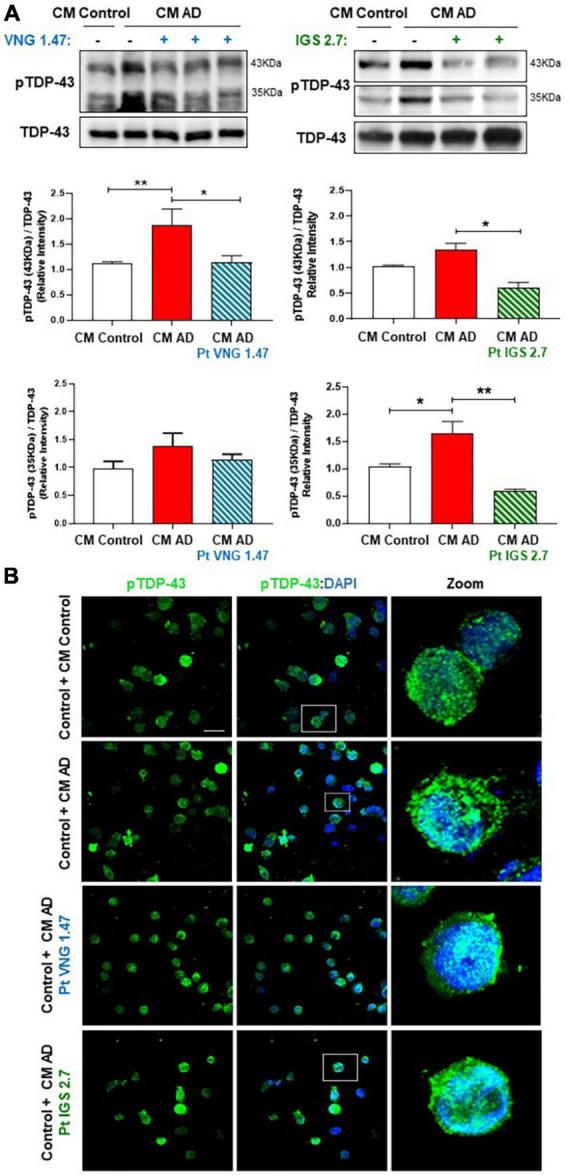
Conditioned medium from AD lymphoblasts pre-treated with kinase inhibitors prevent TDP-43 hyperphosphorylation in healthy lymphoblasts. Control cells were cultured in presence or absence of CM from severe AD cells pre-treated or not with VNG1.47 (10 μM) or IGS2.7 (5 μM) for 72 h. **(A)** Representative immunoblots are shown. Densitometric quantification of pTDP43 (bands of 35 kDa and 43 kDa) was normalized with total TDP-43 levels. Bars are the mean ± SD for each experimental group (2 cell lines for each group) of 4 independent experiments. Bars are the mean ± SD of three independent experiments. Data were assessed by one-way ANOVA and *post hoc* Bonferroni’s analysis (*p < 0.05, **p < 0.01). **(B)** Representative confocal immunofluorescence images of cells stained with anti-TDP-43 antibody (green) and DAPI (blue) are shown. Scale bar, 20 μm. Magnified cells from images are shown on the right panels for better visualization. Fluorescence intensity quantification was performed using Image J software in at least 4 fields of view (A.U, arbitrary units).

Finally, we analysed the phosphorylation status of TDP-43 in these CM studies. TDP-43 phosphorylation was shown to be higher in healthy cells exposed to CM from AD lymphoblast than in those exposed to CM from healthy cells in both, the full-length protein and in the 35 kDa fragment (Figure 4A). However, control cells exposed to ptCM from AD with VNG1.47 or IGS2.7 (Figures 4A, B) showed a significant decrease in TDP-43 phosphorylation levels compared to untreated cells. Therefore, these kinase inhibitors are able not only to recover TDP-43 homeostasis intracellularly but also to prevent propagation of TDP-43 pathology.

### 3.3. Intracellular transport study in U2OS treated with the conditioned medium from AD lymphoblasts without and with VNG1.47 and IGS2.7 treatment

In previous studies, we have shown that human osteosarcoma U2OS cells treated with the conditioned medium from lymphoblasts from severe AD patients also showed increased phosphorylation and mislocalization of TDP-43 (Cuevas et al., 2022). Because of that, we selected this cellular model, with greater cytoplasm area than immortalized lymphocytes, to study a dynamic function of the receiving cells to explore whether this transmission of pathology altered other key cellular functions beyond TDP-43 homeostasis. Therefore intracellular transport deficits were studied, given the relation between these two biological mechanisms (Wood et al., 2021). We took advantage of the previously designed and validated peptide probes to monitor intracellular transport. Specifically, we monitored anterograde transport mediated by kinesin (KBP) using a probe containing the cyanine fluorophore Cy5. The Cy5-KBP probe was successfully used in a SARS-CoV-2 drug discovery program (Oliva et al., 2022), and was selected here to provide more insights on the relation among TDP-43 aggregates and intracellular transport. The behavior of the particles was described by determining the mean track displacement.

**FIGURE 5:**
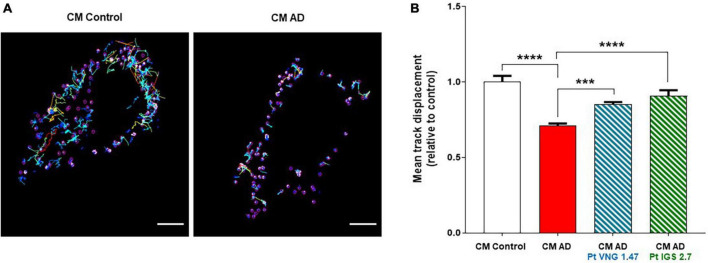
Cy5-KBP trajectories analyzed in U2OS cells. **(A)** Treated with CM from lymphoblasts from healthy subjects and AD patients; **(B)** and with CM or ptCM from AD patients treated with VNG1.47 or IGS2.7. Mean track displacement was analyzed using ImageJ and related to control. Data represent the mean ± SEM of two independent experiments with three different fields analyzed. One-way ANOVA analysis followed by Bonferroni *post hoc* test was performed to determine significance (***p < 0.001, ****p < 0.0001).

U2OS cells were treated with conditioned medium from healthy lymphoblasts and lymphoblasts from two different severe AD patients for 72 h. A significant reduction on the average displacement of Cy5-KBP particles was found compared to U2OS treated with conditioned medium from healthy controls, showing a deficit in the intracellular transport in AD samples (Figure 5A). Furthermore, tracking analysis of Cy5-KBP revealed that either ptCM from AD cells treated with VNG1.47 or IGS2.7 reverts the intracellular transport deficits in U2OS (Figure 5B).

## 4. Discussion

Increasing amount of evidence indicates that TDP-43 pathology may play an important role in AD. TDP-43 inclusions have been found in approximately 50% of necropsies, most often in severe cases of AD, suggesting that TDP-43 can influence AD pathology and neurodegeneration. The presence of TDP-43 pathology in AD brain has been associated with greater memory loss and brain atrophy.

To date no effective treatment for AD exists, pre-clinical pharmacological approaches aimed at blocking disease progression had been focused at the clearance of extracellular deposits of β-amyloid, formation of intracellular neurofibrillary tangles, or preventing inflammation and oxidative stress among other processes (Yiannopoulou and Papageorgiou, 2020). On the basis of recent findings pointing out to an additional pathogenic role of TDP-43 in AD, either by itself or by interacting with β-amyloid plaques and neurofibrillary tangles formation (Meneses et al., 2021), TDP-43 can be considered as novel therapeutic target for AD disease with comorbid TDP-43 pathology (Latimer and Liachko, 2021).

In consonance with the fact that AD is not only a brain disorder but also presents several peripheral and systemic abnormalities (Wojsiat et al., 2015; Esteras et al., 2016), we recently reported alterations in TDP-43 homeostasis, including increased TDP-43 phosphorylation, truncation and cytoplasmic accumulation in immortalised lymphocytes derived from AD patients. Moreover, we observed an enrichment of a 25 kDa TDP-43 fragment in the extracellular medium from AD cells, and found that conditioned medium from lymphoblasts of severe AD induced TDP-43 pathology in healthy cells (Cuevas et al., 2022). Here, we report that targeting TDP-43 phosphorylation with different kinase inhibitors seems to stop disease propagation. We present our results of the treatment of control and AD lymphoblasts with VNG1.47 and IGS2.7, two in-house small molecules, inhibitors of TTBK1 and CK1, respectively. These kinases are known to phosphorylate TDP-43 *in vivo* and *in vitro* (Kametani et al., 2009; Liachko et al., 2014).

We have previously demonstrated the usefulness of lymphoblasts derived from patients suffering from neurodegenerative diseases to study pathogenic mechanisms, and to perform preclinical evaluation of potential drug candidates. In particular, we reported the ability of CK1, CDC-7 and TTBK1 inhibitors to restore TDP-43 homeostasis in lymphoblasts derived from ALS and FTLD-TDP patients (Alquezar et al., 2016; Martinez-Gonzalez et al., 2020; Nozal et al., 2022).

Both VNG1.47 and IGS2.7 efficiently reduced TDP-43 hyperphosphorylation in severe AD lymphoblasts, and restored the nuclear/cytosolic TDP-43 ratio to values similar to those observed in control lymphoblasts. Interestingly both compounds were able to significantly reduce the number of cells with TNT-like structures, characteristic of lymphoblasts from severe AD patients (Cuevas et al., 2022). These structures were found to co-localize with TDP-43 aggregates, suggesting that they can participate in the intercellular dissemination of TDP-43 pathology.

It was previously shown that a fragment of approximately 25 kDa was secreted to the extracellular medium from AD lymphoblasts have prion-like characteristics (Cuevas et al., 2022). Indeed, conditioned medium from the severe AD cultures induced not only TDP-43 pathology (increased phosphorylation and cytoplasmic accumulation of the protein), but also cytoskeletal abnormalities with increased formation of actin protrusions. Our data show the ability of VGN1.47 and IGS2.7 for blocking the cell-to-cell propagation of the disease. Together these results revealed the potential usefulness of these compounds in a future treatment for AD.

Considering that AD-associated changes detected in peripheral cells from patients might not fully reflect alterations of AD brain, it may be worthwhile to mention that at the doses used in this work, VNG1.47 and IGS2.7, were able to prevent ethacrynic-induced cell death in neuroblastoma SH-S5Y5 cells, and exerted neuroprotective effects in a murine model of ALS (Martinez-Gonzalez et al., 2020; Nozal et al., 2022). These observations further support for the utilization of lymphocytes from patients to evaluate the modulation of molecular pathogenic mechanisms.

Protein aggregates had been associated with impaired intracellular transport in neurodegenerative diseases (Wood et al., 2021). Thus, we considered interesting to evaluate whether altered phosphorylation and truncation of TDP-43 protein could participate in the impaired kinesin-mediated intracellular transport in AD (Morotz et al., 2019). Intracellular transport was monitored in human osteosarcoma U2OS cells after inducing TDP-43 pathology by treating them with conditioned medium from severe AD cells, as described previously (Cuevas et al., 2022). Our data support the involvement of TDP-43 in regulating intracellular transport. Interestingly, the inhibition of TDP-43 phosphorylation by both VNG1.47 and IGS2.7 ameliorated the cytotoxic effect of aggregates, restoring normal rates of cellular transport.

## 5. Conclusion

TDP-43 plays a key role in several neurodegenerative diseases including AD. The present results indicate that inhibiting TDP-43 phosphorylation in human cell models of AD, by targeting TTBK1 or CK1 is enough not only to recover nuclear TDP-43 localization, but also to reduce the cytotoxic effect of protein aggregates in intracellular transport. Moreover, and probably biologically relevant, the inhibition of these two kinases avoid the cell-to-cell disease dissemination. Taken together, these small, brain-permeable molecules, VNG1.47 and IGS2.7 can be considered good drug candidates for the treatment of AD with comorbid TDP-43.

## Data availability statement

The raw data supporting the conclusions of this article will be made available by the authors, without undue reservation.

## Ethics statement

The studies were conducted in accordance with the local legislation and institutional requirements. Written informed consent for participation was signed from the participants or the participants’ legal guardians. All study protocols were approved by the Ethic Committee of Clinical Investigation of the Hospital 12 de Octubre (CEIC02506) and the Spanish National Research Council Institutional Review Board (15 March 2007).

## Author contributions

ÁM-R, AM, and CG: conceptualization. LM-G, EC, and CT-B: methodology. LM-G, EC, CT-B, VP, and VN: investigation. ÁM-R and AM: resources. AM, LM-G, and VN: writing—original draft preparation. EC, CT-B, LM-G, CG, VP, ÁM-R, and AM: writing—review and editing. AM and VP: funding acquisition. All authors have read and agreed to the published version of the manuscript.
